# Mechanisms of the effect of fertility policies on the labor-capital income gap

**DOI:** 10.1371/journal.pone.0301347

**Published:** 2024-04-26

**Authors:** Wei Cui, An-Wei Wan, Yuan Zheng

**Affiliations:** 1 School of Economics and Management, Nanjing Institute of Technology, Nanjing, R.P. China; 2 Business School, Hohai University, Nanjing, R.P. China; UCL: University College London, UNITED KINGDOM

## Abstract

This paper investigates the impact mechanism by which an incentive-based fertility policy may reduce the labor income share. First, the specific paths through which this impact mechanism is realized are analyzed using the production function. It is found that an incentive-based fertility policy triggers high savings, which implies more, cheaper, and more readily available capital to be invested in production. A distribution system that earns income based on factor contributions results in more gains for capital than labor, i.e., a lower share of labor income and a wider income gap between labor and capital. Second, the impact mechanism includes three theoretical hypotheses. They are that an encouraging fertility policy is negatively related to labor income share; this relationship is valid provided that the study subject is in a closed economy; and that capital intensification is a mediator variable of fertility policy affecting labor income share. Finally, to further corroborate the impact mechanism in this paper, a Hansen threshold panel model is applied to verify that the effect of fertility policy on labor income share has a threshold effect. This indicates that the effect of the former on the latter changes significantly before and after the change in fertility policy, confirming the existence of an impact mechanism. The established literature has paid little attention to the impact of incentivised fertility policies on the labour income gap. Using capital intensification as the mediating variable, this paper demonstrates the existence of the former effect on the latter. In view of this, under the encouraged fertility policy, this paper proposes specific measures to enhance the labor income share in order to narrow the income gap between labor and capital.

## Introduction

**Fig 1:**
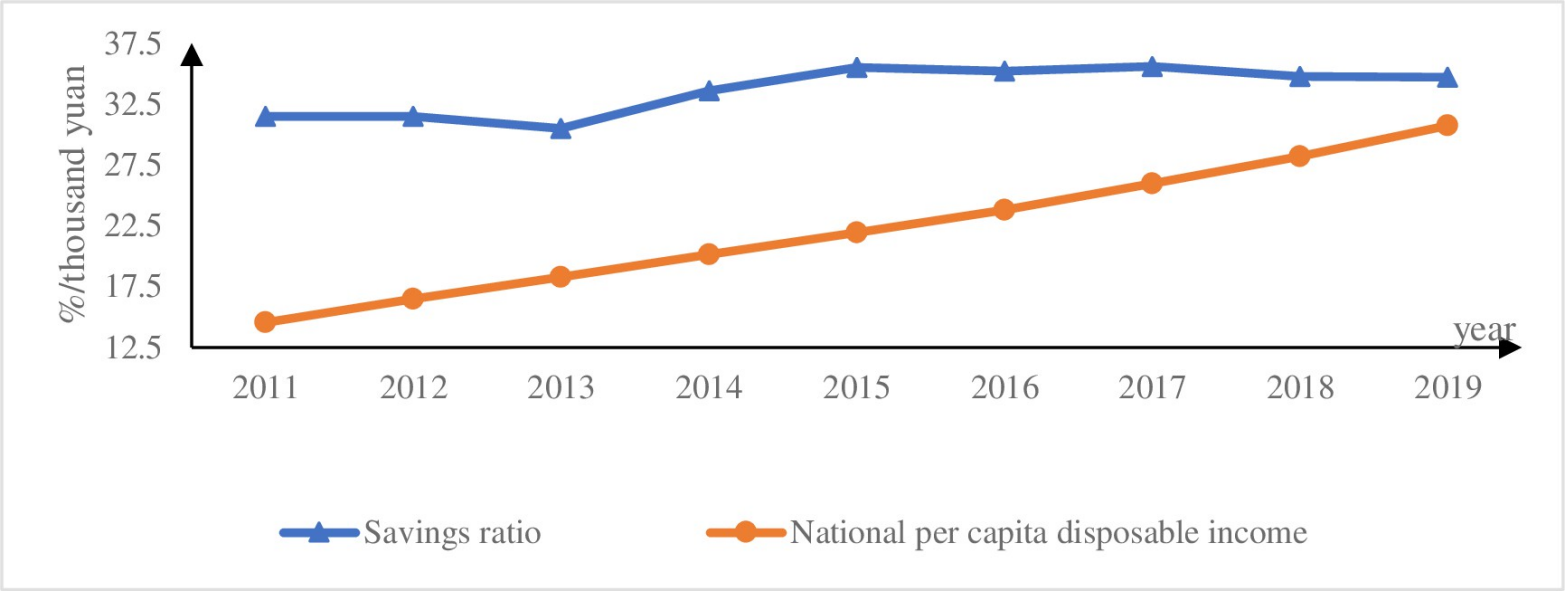
Change in savings rate and national income per capita from 2011 to 2019.

**Fig 2:**
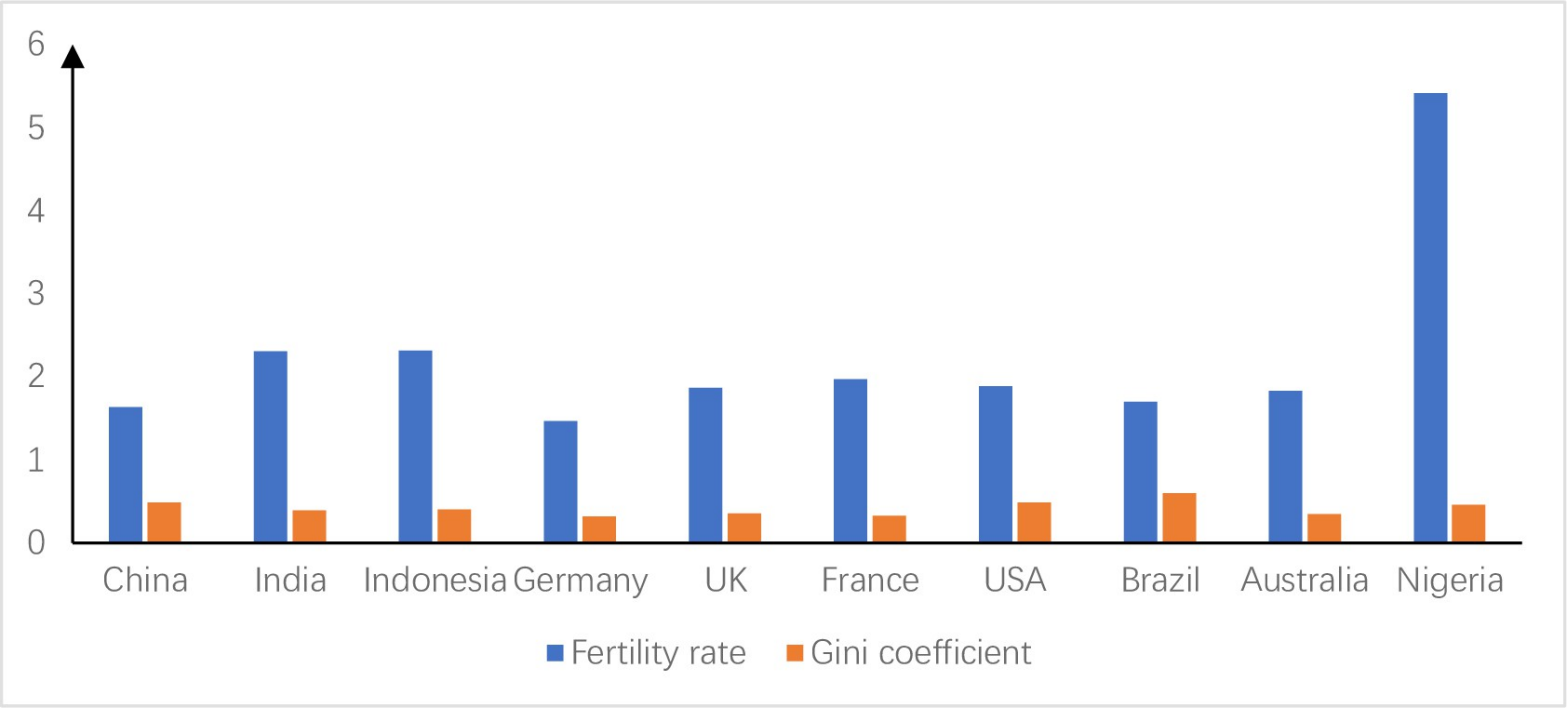
Fertility rates and Gini coefficients for major countries on all continents.

Based on the factor-based income distribution system, the relaxation of China’s fertility policy may widen the income gap between capital owners and labor owners, i.e., the income gap between labor and capital. Although China still maintains the inertia of "not wanting to have children" after the easing of the fertility policy in 2013, the inertia of negative population growth may be reversed as the policy is implemented [1]. Although China’s central government has stipulated that there is no uniform timetable for the implementation of this policy across the country, it is not advisable for provinces and regions to implement it too late. Before the end of 2014, the policy was rapidly implemented throughout China. Just as the one-child policy, which was implemented in 1980, has gradually changed the traditional Chinese concept of having many children and being blessed with many children. In view of this, it should be expected that the multi-child fertility policy may reverse the trend of low fertility intention in China in the future, which in turn affects the saving behavior of households. First, families with many children need to prepare more money for education. According to the findings of the "Cost of Education for Children in Chinese Families with Compulsory Education", Chinese families spend 30% of their income on education, and this proportion is increasing by 20% annually, making it the fastest growing household consumption item. China’s Guangming Daily reported the same findings in 2018, and further found that 80 per cent of education spending comes from out-of-school tutoring. The "2017 White Paper on Family Education in China" analyses education consumption from an expenditure perspective. Chinese families spend more than 50 per cent of their annual household expenditure on education. Second, according to the competitive savings perspective, families need to save 35%-50% more for their children’s marriage [2], especially for families at a disadvantage in saving for marriage [3], and this has led to a "marriage tournament" [4]. Third, there are unforeseen medical costs. Although China has achieved universal health insurance, major illnesses and serious diseases are still a potentially heavy burden for families, for which they need to set aside a considerable amount of precautionary savings. At the macro level, savings of all households form a source of investment for society as a whole [5, 6]. Relevant data also corroborate that incentivised fertility policies may boost savings rates, as shown in Fig 1. Fig 1 compares the changes in China’s savings rate and national income per capita from 2011–2019 in China. China introduced the selective two-child policy in November 2013. In order to compare the savings rate before and after the policy change, this paper starts counting the values of the relevant indicators from 2011. Because the novel coronavirus epidemic occurred at the end of 2019, the relevant data were affected by external shocks, so the end of the statistics is in 2019. As can be seen in Fig 1, changes in the savings rate are weakly correlated with changes in income, but may be strongly correlated with changes in fertility policy. The savings rate was stable at around 31 per cent before the fertility policy change in November 2013, and in comparison, there was a significant increase in the savings rate after the implementation of the incentivised fertility policy. Thus, changes in fertility policy may affect income distribution through savings rates, ultimately leading to changes in income disparity. However, changes in fertility rate and income gaps in other countries around the world do not conform to this pattern, as shown in Fig 2. Fig 2 selects the major populous countries on all continents of the world in 2019 for the study and compares their fertility and income disparities, with income disparity measured by the Gini coefficient. The comparison reveals that India and Indonesia have high fertility rates after Nigeria, but the Gini coefficient is in the range of 0.3–0.4, which is in the reasonable range for income disparity. In contrast, Brazil, the United States and China have higher Gini coefficients despite having lower fertility rates, with Brazil even reaching over 0.6. Therefore, the study in this paper is limited to the cultural context of China only.

With the significant increase in household savings, it leads to an increase in the amount of physical capital inputs in social production. In comparison, the amount of labor input grows less rapidly than physical capital. The distribution of income according to factor contributions and the widening gap between labor and capital income may stimulate antagonism among different income classes and cause social conflicts, yet few studies have focused on this issue. In order to curb the widening income gap, the mechanism should be clarified, followed by finding the specific reasons for the widening gap between labor and capital income due to the relaxation of fertility policy, and taking corresponding countermeasures. Existing research lacks the expectation that incentivised fertility policies may reverse the low fertility trend in the future, and thus pays insufficient attention to the ensuing decline in the labour income share. In view of this, this paper digs deeper into the mechanism by which the relaxation of fertility policy affects the labour income share and proposes a scientific solution to increase the labour income share and mitigate the widening labour income gap.

The subsequent structure of the thesis is organised as follows: a literature review in Section 2, an analysis of the theoretical mechanisms in Section 3, an empirical test of the theoretical mechanisms in Section 4, and conclusions and policies in Section 5.

### Effect of fertility rate on physical capital accumulation

Fertility changes may alter the demographic structure, which in turn affects the accumulation of physical capital. After the baby boom, the decline in fertility rate instead yields a demographic dividend [7–9], due to the decline in birth rate, which reduces the cost of raising minors. The slowdown in population growth is followed by an increase in capital efficiency [10]. However, as life expectancy increases, an aging society may be entered if fertility rates continue to decline.

Aging societies trigger saving effect and burden effect. There are two main types of saving effects, one is the weakening of parents’ precautionary demand for their children due to longer life expectancy [11], which together with the increase in infant and child survival rates also strengthens the positive precautionary savings rate [12]. A decline in fertility also leads to labor shortages [13, 14] and therefore an increase in the number of working years, which also increases the incentive for rational people to save and an increase in physical capital accumulation [15, 16]. A decline in fertility may also see a decline in the savings rate and physical capital accumulation, i.e., a burden effect. This is because aging also implies an increase in the burden of support for the elderly, requiring more pension and health insurance payments, leading to a decrease in the savings rate [17, 18] and possibly offsetting the savings effect [19, 20]. Some scholars have also argued that the burden effect is exaggerated because as population size declines, capital per capita will rise [21] and there will be no significant decline in the demographic dividend.

From the above analysis, it is clear that fertility has a significant effect on the accumulation of physical capital. Because China has implemented the one-child policy for more than 30 years, most of the literature analyzes the impact of fertility decline on savings, and less often analyzes the change in savings rate and the impact on physical capital in the context of the current incentive fertility policy and the change in the labor-capital income gap triggered.

### The impact of material capital accumulation on income distribution

The change in fertility rate triggers a change in physical capital accumulation and a corresponding change in individual income distribution, which manifests itself in two ways: a sense of equity and a relative income gap.

Views that physical capital accumulation affects the sense of fairness in income distribution are divergent. One view is that with the accumulation of physical capital, individuals’ sense of fairness in income distribution increases [22], especially the stronger this sense of fairness is with the increase of individuals’ physical capital [23]. Moreover, the much-publicized decline in the urban-rural income gap is also achieved by increasing the accumulation of productive household fixed assets, which enhances the individual’s sense of fairness [24]. These ideas can be verified with the help of the Human Development Index HDI published by the United Nations: physical capital enhances individuals’ quality of life, life achievement and security [25]. However, there is a small number of contrary studies that argue that the increase in physical capital instead reduces the sense of individual equity [26, 27].

Physical capital affects the relative income gap through skills [28]. As the dominant role of technology in production increases, physical capital is increasingly dependent on labor force skills [29–33]. The main manifestation of this is the continuous accumulation of physical capital and the differentiation of labor with different skills [34], the materialization of technological progress is prominent, and the complementarity of "capital-skill" is obvious [35, 36]. After Griliches proposed the theory of "capital-skill" complementarity [37], the academic community began to realize that there are differences in the elasticity of substitution between factors of production [38]. According to this theory, the higher the amount of physical capital input, the higher the demand for skilled personnel and the lower the demand for unskilled personnel [39], which raises the skill premium [40] and widens the income gap between workers with different skills, and numerous studies have verified this argument [41–46].

### Literature related to research methodology

The first is the method of studying the effect of fertility on capital accumulation. Akira constructed a Cobb Douglas production function to analyze the effect of population increase on changes in per capita capital stock [47]. However, this approach ignores dynamic changes, so most studies adopt two-period overlapping generation model or overlapping generations model [48, 49]. The second is the research method of capital accumulation affecting income disparity. The effect of the former on the latter is based on the dependence of capital on labour skills, i.e. capital-skill complementarity [37]. This theory suggests that if the scale of investment in physical capital increases, then the demand for skilled labour rises and the demand for unskilled labour falls [50]. Initially this research was concerned with testing this theory [51]. As statistics became more robust and econometric theory developed, this theory began to focus on the income distributional effects of capital-skill complementarities. The significance of this is that physical capital accumulation reinforces the working hours of skilled labour, increases the ’effective’ skill time of skilled labour and reduces the ’effective’ skill time of unskilled labour, thereby increasing the marginal output of skilled labour and decreases the marginal output of unskilled labour, which ultimately leads to a widening of the income gap [52–54]. The main methods used in the study are the least squares method and the CES production function.

**Fig 3:**
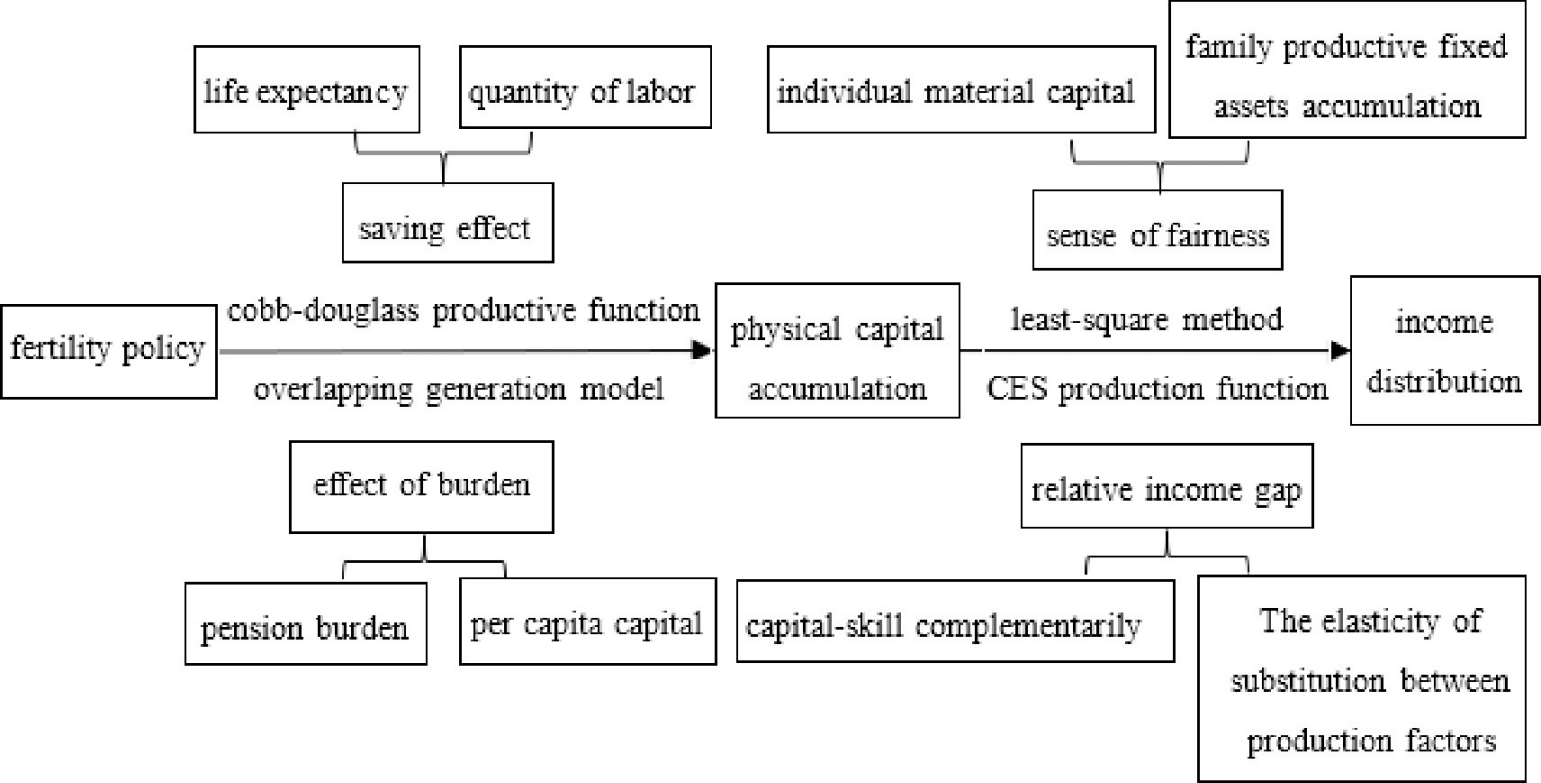
Literature research.

The above literature study is summarized in Fig 3.

There have been studies that have analyzed in depth the relationship between fertility policy and physical capital input, physical capital and individual income gap, which have promoted the development of income distribution theory, but there are three problems that need to be analyzed in depth as follows. First, it ignores that the effects generated by changes in fertility policy can affect the labor-capital income gap through physical capital, which may result in an income distribution overly favoring owners of physical capital and leading to an increase in the income gap between labor and capital. Second, the mechanism of the impact of fertility policy on the income distribution between labor and capital is unknown, resulting in the lack of scientific secondary or tertiary distribution measures to cope with fertility policy changes and the inability to effectively mitigate the widening trend of the income gap between labor and capital. Thirdly, studies have focused on the impact of changes in physical capital on the income gap between laborers with different skills, ignoring the labor-capital income gap, which is also the main cause of income inequality. Fourthly, the lack of a methodology that incorporates fertility policy, physical capital and labour-income gaps into the same theoretical model has resulted in the absence of a theoretical mechanism for the relationship between the three. In view of this, this paper analyzes the mechanism by which fertility policy affects the income distribution between labor and capital with the help of production function, which includes: the intermediary mechanism of capital intensity as a mediator variable, the mechanism of physical capital investment excluding foreign capital and the threshold mechanism of fertility policy, and accordingly finds the scientific path to suppress the widening of the income gap between labor and capital.

## Theoretical mechanism analysis

The fertility policy variables are set to *ξ*. *ξ* = 1 denote the one-child policy, where a couple is allowed to have only one child, and *ξ*>1 denotes the encouraged fertility policy, where a couple is allowed to have more than one child. For example, if China introduces a three-child policy in May 2021, where a couple is allowed to have three children, then *ξ* = 3. Theoretical and data studies have shown that the savings rate tends to increase as the number of children in a family increases [55, 56]. Let Z be the factors that affect the saving rate besides the fertility policy, such as the market interest rate and total income. The savings rate function is denoted as

$$s_{\xi }=S(\xi ,Z)$$ (1)

The function is a monotonically increasing function, i.e., an incentive-based fertility policy will raise the savings rate. This is because during the period of the family-planning policy, a couple who has more children than the policy limit will have to pay high social maintenance fees and even be fired from their workplace, which greatly reduces the wealth of the family and lowers the overall savings rate of the society [57]. Moreover, raising one child is less costly than raising multiple children. In addition, the rising level of per capita income in China, the increasingly prevalent concept of over-consumption, the improving social security system [58], the gradually increasing awareness of financial management, and the conversion of savings into different risky investments have all contributed to the decrease of the social savings rate. In comparison, the relaxation of the family-planning policy helps to raise the savings rate. Because families with many children pay more for health care, education and housing than families with only one child, moreover, these expenditures account for a larger share of total household expenditures, so families with many children need to have a higher savings rate.

The impact of China’s incentive-based fertility policy on the labor-capital income gap is analyzed with the help of the production function, as in Eq (2). Because this paper compares the income of capital and labor owners in society as a whole, the income of labor can be represented by the labor income share and the income of capital owners by the capital income share, and any relative change in the income share of either factor will lead to a change in the income gap. In view of this, this paper chooses the labor income share to compare the income gap between the two factors. If the labor income share is significantly higher, then the income of the labor factor increases; conversely, the income of the capital factor increases. It is assumed that the economy produces two factors of input capital (*K*) and labor (*L*) and the elasticity of substitution of these two factors in the production function is constant.


$$Y=A{\left[\rho K^{\left(\theta -1\right)/\theta }+(1-\rho )L^{\left(\theta -1\right)/\theta }\right]}^{\theta /(\theta -1)}$$ (2)

*Y* in Eq (2) is the total social output, *K* and *L* are the stock of capital and labor invested in the production process, respectively, A is the technological progress, *ρ*∈(0,1) is the share of capital income, and *θ*∈(0,∞) is the elasticity of substitution between capital and labor. Both sides of Eq (2) are divided by *L* at the same time to obtain Eq (3).


$$y=A{\left[\rho k^{\left(\theta -1\right)/\theta }+(1-\rho )\right]}^{\theta /(\theta -1)}$$ (3)

From Eq (3), we obtain the social output per capita y and the capital intensity *k* (*k = K/L*). In a perfectly competitive market structure, the return to labor is *ω*, and the return to capital is *γ*. The profit maximization condition is that the marginal output of labor and capital factors is equal to the factor price, i.e.


$$\omega =A(1-\rho ){\left[\rho k^{\left(\theta -1\right)/\theta }+(1-\rho )\right]}^{1/(\theta -1)}$$ (4)

$$\gamma =A\rho {\left[\rho +k^{\left(1-\theta \right)/\theta }(1-\rho )\right]}^{1/(\theta -1)}-1$$ (5)

The labor income share (*LS*) is defined in Eq (6).


$$LS=\frac{\omega L}{\omega L+\gamma K}=\frac{\omega L}{Y}=\frac{\omega }{y}$$ (6)

From Eqs (3), (4) and (6), we have

$$LS=\frac{\left(1-\rho \right)}{\rho k^{\left(\theta -1\right)/\theta }+(1-\rho )}$$ (7)

Eq (7) shows that the labor income share is related to capital intensification, capital income share, and labor elasticity of substitution coefficient. According to Horioka and Terada-Hagiwara’s study [59], there is a positive relationship between the social capital intensity and the aggregate savings rate. This is because the amount of investment in an economy is determined by the savings rate, and the higher the total savings rate, the lower the difficulty of lending. The capital intensity, i.e., the amount of investment per capita, k, is similarly positively correlated with the savings rate. Provided that the marginal output of capital is determined, the higher the aggregate savings rate, the more the society demands for capital, as shown in Eq (8). And, combined with Eq (1), it is clear that the capital intensity k is also a function of fertility policy *ξ*.


$$k=k(s_{\xi }),\;s_{\xi }>0$$ (8)

Based on Eqs (1) and (8), the partial derivative of the labor income share in Eq (7) with respect to the fertility policy variable *ξ* yields Eq (9)

$$\frac{\partial LS}{\partial \xi }=\frac{\partial LS}{\partial k}\cdot k_{s}\cdot s_{\xi }=-(\frac{\theta -1}{\theta })[\frac{\rho (1-\rho )k_{s}\cdot s_{\xi }}{k^{1/\theta }\cdot {\left(\theta k^{\theta -1/\theta }+(1+\rho )\right)}^{2}}]$$ (9)

From Eq (9), because $\frac{\rho (1-\rho )k_{s}\cdot s_{\xi }}{k^{1/\theta }\cdot {\left(\theta k^{\theta -1/\theta }+(1+\rho )\right)}^{2}}>0$, the effect of fertility policy changes on labor income share is determined by the factor elasticity of substitution *θ*. If the two input factors are substitutes for each other, i.e., *θ*>1, then the result of Eq (9) is less than 0, i.e., encouraged fertility reduces the labor income share and widens the income gap between labor and capital; if the two input factors are complementary, i.e., 0<*θ*<1, then the result of Eq (9) is greater than 0, i.e., encouraged fertility policy helps raise the labor income share and reduces the income gap between labor and capital. If the two types of input factors are neither complementary nor substitution, i.e., *θ* = 1, then the income gap between labor and capital is not affected by the fertility policy.

For most enterprises in China, labor and capital are often substitutes for each other [60]. Therefore, with the implementation of the "separate two-child policy", "comprehensive two-child policy" and "liberalization of the three-child policy", the labor income share tends to gradually decrease, i.e., the gap between labor The gap between labor and capital income is gradually widening.

In summary, the mechanism of the effect of fertility policy on the income gap between labour and capital can be derived. In a closed economy, fertility policy affects the income gap between labour and capital with physical capital investment as a mediator variable. As fertility policy is relaxed, the labour-capital income gap widens and a threshold effect may emerge at the point of policy change.

## Mechanism testing

### Data selection, descriptive statistics and model setting

Because the fertility policies of ethnic minorities are relatively lenient compared to those of Han Chinese, the impact of fertility policies on labour income shares in cities with a concentration of ethnic minorities may differ from other regions. Therefore, grouping by province and region tends to obscure this difference between cities with a concentration of ethnic minorities and other cities, losing some important information and failing to highlight statistical regularities. In view of this, this paper selects the data related to 293 prefecture-level cities in mainland China for mechanism validation, with a time span of 2003–2019. Due to the large time span, it is inevitable that the phenomenon of missing statistics and changes in city administrative divisions occurs, for example, the relevant data of six cities in Tibet are missing, and changes in city administrative divisions occur after 2010 in Anhui Province, Hainan Province, Guizhou Province, Qinghai Province and Xinjiang Uygur Autonomous Region. Moreover, the companies selected in this paper are listed companies that have been in continuous operation during the survey time, and the missing control variables and ST companies are excluded. After eliminating the above missing data, this paper identifies 3,166 listed firms in 285 prefecture-level cities, of which the sample size is 3,015 for domestic firms and 151 for wholly foreign-owned firms, and the sample in this paper covers 97% of the cities in China.

Based on the mechanism analysis, the labour income share (*LabS*) was used as the dependent variable to measure the labour-capital income gap, which follows the measure of Cui et al. [60]. The labour income share is measured in two ways. At the macro level, labour income share refers to the ratio of workers’ income to GDP, and is calculated as follows: labour income share = total workers’ compensation/(net production tax + operating surplus + depreciation of fixed assets + total workers’ compensation); at the micro level, labour income share refers to the proportion of workers’ compensation to the value added of enterprises. Because the relevant indicators can be obtained from the "China Industrial Enterprises Database" and the "Listed Companies Database", but the indicators in these two databases are not exactly the same, so the specific calculation methods are also different. If the data comes from "China Industrial Enterprises Database", then the compensation of workers in the enterprise = total wages payable + total welfare payable. Moreover, according to the factor income method and the factor cost method, there are two ways to calculate the value added of an enterprise. Firstly, the value added of the enterprise = depreciation + labour compensation + operating surplus + net production tax; secondly, the value added of the enterprise = depreciation + labour compensation + operating surplus. If the data comes from the "Listed Company Database", then the compensation of workers of the enterprise is selected from "Cash paid to and for employees" in the cash flow statement or "employee remuneration payable" in the balance sheet. The value added of the enterprise is the total operating income or the total assets at the end of the period, or the sum of the compensation of workers, operating profit and depreciation of fixed assets. This paper uses the Cui et al. measure of labour income share, i.e. the ratio of employee compensation to firm value added. Employee compensation is derived from "cash paid to employees" in the cash flow statement; value added of the enterprise = operating income—operating costs + employee compensation + depreciation of fixed assets. Li et al. [61] used the same method in calculating the share of labour income.

The dependent variable, fertility policy (*BirP*), was chosen as a dummy variable. China introduced the "the selective two-child policy" in November 2013, which was the starting point for fertility policy easing since the implementation of the one-child policy, followed by the further implementation of the "universal two-child policy" in early 2016, and the fertility policy encourages of three-child births from 2021. Based on this, the fertility policy variable is zero until 2014 and one thereafter. fertility policy affects the labour income share by changing the savings rate. The savings rate is equal to the capital intensity (*CapI*), so this paper chooses the capital intensity as the mediator variable to test the effect of fertility policy on the labour income share. The capital stock is calculated as the ratio of a firm’s capital stock to its average headcount.

The control variables were selected with reference to the study by Liu and Wang et al. [62]. First, technological progress (*TECH*). At the firm level this indicator is the ratio of new product output to total industrial output. Technological progress can be either labour-biased or capital-biased, helping to raise the labour income share if it is the former, or possibly lowering it if it is the latter. Second, the length of time the firm (*LTF*) has been in existence. As a firm develops and grows, the share of factor income distribution changes. Third, the size of the firm (*SF*), which is the logarithm of the firm’s total assets. Capital is a major component of a firm’s total assets compared to labour. The larger the enterprise and the stronger the capital, the lower the share of labour income is likely to be. Fourth, the interest rate of the business (*IR*), which is measured as the ratio of interest payable by the business to its total assets. The higher the cost of borrowing for the enterprise, the less revenue is available for distribution and the lower the share of labour income is likely to be. Fifth, the firm’s exports of products (*FEP*). This indicator is expressed as a proportion of the enterprise’s export sales to total sales. Sixth, the ownership of the enterprise (*OE*), expressed as a dummy variable. Generally, the enterprise is publicly owned and the distribution of the enterprise’s earnings is biased towards labour. If the enterprise is publicly owned, then the value is 1, otherwise it takes the value of 0.

To exclude unobservable industry characteristics, regional characteristics and time characteristics that do not vary over time and to prevent endogeneity, industry fixed effects *λ*_*i*_, regional fixed effects *λ*_*c*_ and annual fixed effects *λ*_*t*_ are also placed in the model. Descriptive statistics for the above variables are shown in Table 1.

**Table 1:** Descriptive statistics.

Variable	observation number	Mean	Min	Max	Median	SD
*LabS*	27403	0.3172	0.2874	0.3551	0.2992	0.2011
*CapI*	27403	4.2351	3.9447	4.7965	4.4526	1.4482
*TECH*	27403	0.0400	0	0.0450	0	0.1510
*LTF*	27403	8.3312	2.3121	12.3371	6.2052	9.4472
*SF*	27403	11.3534	6.5875	13.5572	10.2913	2.7742
*IR*	27403	0.0243	0	0.0295	0.0212	0.0334
*FEP*	27403	0.3843	0	0.8756	0.4667	0.2412

Data source: calculated.

Based on the above settings of dependent, independent and control variables, a model of the impact of fertility policy on labour income share can be obtained as in Eq (10).


$$LabS_{ict}=\beta_{0}+\beta_{1}BirP_{ict}+\beta_{2}Z_{ict}+\lambda_{i}+\lambda_{c}+\lambda_{t}+\varepsilon_{ict}$$ (10)

Z denotes the control variable, the subscripts *i*, *c* and *t* denote firm, city and year respectively, and *ε*_*ict*_ denotes random error.

### Mechanism test results

To avoid the problem of heteroskedasticity, the mechanism test uses a linear regression model based on the parameter covariance matrix estimator [63]. Because the mechanism assumes that the economy is in a closed state, the sample is chosen from 3015 domestic firms. Column (1) of Table 2 shows the results of a mechanistic test of the effect of fertility policy on labour income shares in the absence of control variables. Column (2) introduces control variables. Columns (3)-(6) are robustness tests, excluding cities with a high concentration of ethnic minorities, anomalous sample points, the Gini coefficient replacing labour income shares and excluding other policy effects respectively.

**Table 2:** Mechanism test and robustness test results.

	(1)	(2)	(3)	(4)	(5)	(6)
	Mechanism Test		Excluding cities with a high concentration of ethnic minorities	Removal of abnormal sample points	Gini coefficient replacement income share	Exclusion of other policy effects
*BirP*	-0.3532***	-0.1103**	-0.0588***	-0.0430***	0.0461***	-0.0311***
	(0.0561)	(0.0453)	(0.0033)	(0.0062)	(0.0057)	(0.0035)
*TECH*		-0.0362*	-0.0581*	-0.0372**	-0.0442***	-0.0441**
		(0.0377)	(0.0036)	(0.0055)	(0.0057)	(0.0071)
*LTF*		0.0031**	0.0061***	0.0027**	-0.0051***	-0.0068 (0.0052)
		(0.0017)	(0.0151)	(0.0071)	(0.0046)	
*SF*		-0.0183**	-0.0293*	-0.0411*	0.0677**	-0.0413***
		*(0.0218)	(0.0261)	(0.0104)	(0.0325)	(0.0056)
*IR*		-0.2104**	-0.1944**	-0.2245***	0.3045**	0.2274**
		(0.0391)	(0.0472)	(0.0751)	(0.0052)	(0.0231)
*FEP*		0.0611***	0.0514***	0.0433**	-0.0304**	-0.0522**
		(0.0281)	(0.0392)	(0.0079)	(0.0551)	(0.0604)
*OE*		0.1045***	0.0481***	0.0291***	-0.0637***	-0.0468***
		(0.0551)	(0.0603)	(0.0095)	(0.0304)	(0.0377)
*SP*						0.0272** (0.0870)
*IP*						0.04911*** (0.0394)
*C*	0.2948**	0.4911*	0.3704***	0.3382***	0.4432***	0.5531***
	(0.0079)	(0.0414)	(0.0402)	(0.0801)	(0.0061)	(0.0422)
*ON*	27403	27403	19426	21688	27403	27403
*R* ^2^	0.6315	0.7152	0.5692	0.6392	0.5752	0.6615

Data source: calculated

Note: *, ** and *** denote 10%, 5% and 1% significance levels respectively; standard errors of coefficient estimates in parentheses; *N* denotes not controlled; *Y* denotes controlled. *ON* denotes observation number.

Firstly, the first two columns are tests of the impact mechanism. Comparing columns (1) and (2) reveals that while the estimated coefficient on fertility policy in column (1) is consistent with the theoretical mechanism analysis and holds significantly, the effect on labour income share is significantly larger than that in column (2), probably because the model corresponding to the results in column (1) suffers from endogeneity problems, leading to large regression coefficients.

Secondly,. Column (2) analyses the effect of fertility policy on the labour income share with the inclusion of control variables. The regression results are similar to those in column (1), in that incentivized fertility policies are still inversely related to labour income shares, with higher numbers of births leading to lower labour income shares.

Thirdly, the regression results for the other control variables are also significant, consistent with the findings of the existing literature. Technological progress is negatively correlated with the share of labour income, suggesting that the distribution of earnings from technological progress in China is biased towards physical capital, in line with the findings of Zhang et al. [64]. The positive regression coefficient for the length of firm establishment indicates that the longer the firm is established, the more the distribution of income is biased towards labour. This may be due to the fact that the longer the firm is established, the more professional and experienced veteran employees there are, and the higher the value of their labour. Firm size is negatively related to the share of labour income. This may be because the larger the firm, the greater the amount of physical capital invested and the greater the rate of return on this capital, leading to a lower labour income share. Higher interest payments by firms mean that more profits are used to pay off debt, and labour naturally receives less. Exports broaden the outlets for products and services, expanding the sources of income for firms and raising the share of labour income. The public ownership of enterprises helps to raise the share of labour income. Because publicly owned enterprises are owned by the entire population, the distribution of profits earned is also skewed in favour of labour.

### Robustness test

This paper tests the robustness of the model in four ways. Firstly, cities with a high concentration of ethnic minorities are excluded. Because China’s ethnic minority population is a relatively low proportion of the total population, their fertility policies are relatively lenient in order to slow down the decline in the Population size of ethnic minorities. Even during the strict one-child policy period, when Han families had a strict limit of one child, ethnic minority families in comparison were not affected by this quota and could still have two or more children. Thus, in cities with a high concentration of ethnic minorities, changes in national fertility policies may not have had a significant impact on labour income shares. Therefore, these samples may lead to biased significance tests. The Chinese government has set up autonomous prefectures in cities with a concentration of ethnic minorities. There are a total of 30 autonomous prefectures in China, and after excluding the relevant firms in these autonomous prefectures, the significance of the impact of fertility policy on the labour income share is retested, and the results are shown in column (3) of Table 2.

The results show that the estimated coefficient of the fertility policy variable is -0.0372 with a significance level of 1%. This indicates that excluding cities with a high concentration of ethnic minorities, the effect of fertility policy on labour income shares in the remaining cities remains significant, which reinforces the results of the impact mechanism.

Secondly, the effect of excluding outlier sample cities. The outlier sample cities are those with labour income shares below 10% and above 90%. As the dispersion of the sample values of these cities is too large, it affects the overall statistical pattern of the data and may lead to biased test results. After excluding these sample cities, the data from other cities were re-done for regression analysis. The estimated results are shown in column (4) of Table 2. After excluding the sample of outlier cities, the effect of fertility policy on labour income share is still significantly negative with an estimated coefficient of -0.0312 and is significant at the 5% level of significance, indicating the robustness of the model constructed in this paper.

Thirdly, the robustness of the model is analysed by replacing the labour income share with the Gini coefficient. There is an inverse relationship between the labour income share and the Gini coefficient [63]. The labour income share analyses the inequality between labour income and capital income, while the Gini coefficient analyses the share of different income classes. The higher income brackets tend to own a certain amount of capital and their main source of income is capital investment, compared to the lower income brackets who rely mainly on their own labour in exchange for income. Therefore, the Gini coefficient can, to some extent, replace the share of labour income, and after replacement, revert to the model.

The process of calculating the Gini coefficient for each city is as follows. Most of the cities in the "China Statistical Yearbook" are divided into five groups according to low income, low-middle income, middle income, middle-high income and high income. For the other grouping forms in a few areas, this paper is in accordance with the idea of the quintile method of uniformly categorised into five subgroups. According to the Formula (11) to calculate the Gini coefficient *G*, where *P* is the total population, *W* is the total income, and *W*_*i*_ is the income accumulated to the *i*th group.


$$G=1-\frac{1}{PW}\sum_{i=1}^{n}(W_{i-1}+W_{i})\cdot P_{i}$$ (11)

The estimated results are presented in column (5) of Table 2. The effect of fertility policy on the Gini coefficient is positive at the 1% level of significance, with an estimated coefficient of 0.0331. The regression results remain significant after replacement of the dependent variable, suggesting that encouraging fertility policies widen the labour-capital income gap, which further demonstrates the scientific validity of the impact mechanism in this paper.

Finally, real estate policy (*SP*) and industrial policy (*IP*) have significant effects on income inequality [65, 66]. There are two main ways in which real estate policy affects labour income share. On the one hand, real estate policies affect housing costs. For example, the affordable housing policy reduce housing costs and raise the labour income share. On the other hand, real estate policy regulates the real estate market, curbing the excessive rise in housing prices and reducing the pressure to buy a house, which helps to raise the labour income share. The impact of industrial policy on labour income share has two main aspects. On the one hand, industries supported by industrial policy show a tendency to expand, and in order to meet this tendency, higher incomes are needed to attract more labour, so the labour income share may rise. On the other hand, industrial policy tends to support competitive high-technology firms and firms in strategic emerging industries. As they have stronger market competitiveness and profitability, the labour income share may also rise.The real estate policy and industrial policy select the fixed assets investment and the proportion of urban secondary industry, respectively. As shown in column (6) of Table 2, the negative correlation between incentives for childbearing and the share of labour income remains at the 1% significance level, controlling for other relevant policies, indicating the robustness of the model in this paper.

### Endogeneity test

**Fig 4:**
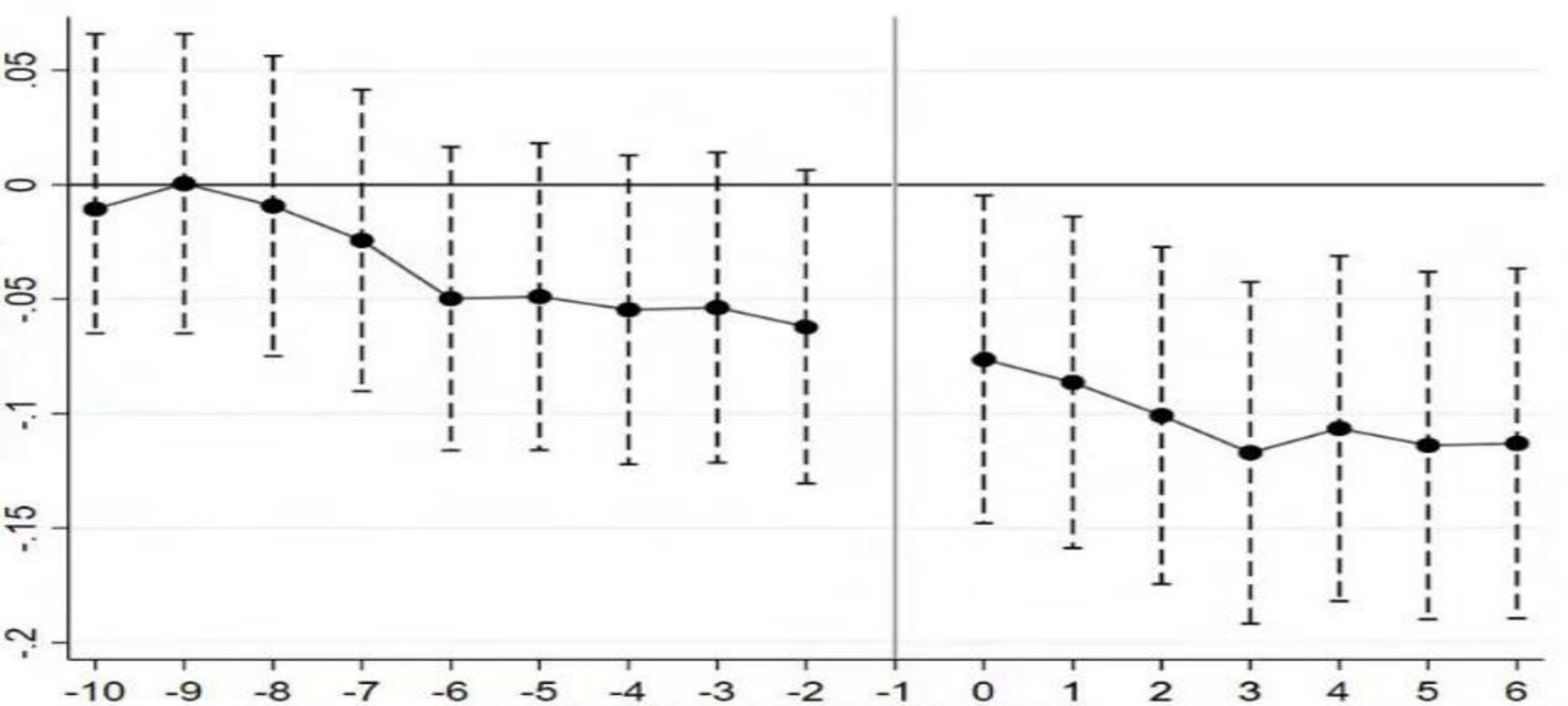
Parallel trend test.

The effect of fertility policy on labour income share may be endogenous, which is tested in this paper using a difference-in-difference model. Minority-aggregated cities are set as the control group and other cities are set as the experimental group, and the test results are shown in Table 3. The results in Table 3 are consistent with those in Table 2, so there is no endogeneity in the effect of fertility policy on labour income share. The premise of the validity of the difference-in-difference model is that the treatment and control groups can satisfy the assumption of parallel trends. That is, before the change in fertility policy, the labour income shares of the treatment group and the control group are consistent in time trend; after the change in fertility policy, they are different. Although the study period of this paper is 2003–2019, there was a change in fertility policy at the end of 2013, so the time period for the parallel trend test can be limited to around 2013. This paper is set to 2010-2019.The results of the parallel trend test are shown in Fig 4, from which it can be seen that the difference-in-difference model passed the parallel trend hypothesis test.

**Table 3:** The impact of fertility policy on the income gap between labour and capital based on the DID model.

	(1)	(2)
*BirP*	-1.2703***	-0.6903***
	(0.2291)	(0.0551)
*TECH*		-0.0425*
		(0.0037)
*LTF*		0.0047***
		(0.0053)
*SF*		-0.0392**
		(0.0514)
*IR*		-0.3306***
		(0.2281)
*FEP*		0.0616**
		(0.0391)
*OE*		0.0481**
		(0.0803)
*C*	0.7751***	0.5517***
	(0.0683)	(0.0427)
observation number	27403	27403
*R* ^2^	0.6833	0.6605

Data source: calculated

Note: *, ** and *** denote 10%, 5% and 1% significance levels respectively; standard errors of coefficient estimates in parentheses.

### Dynamic analysis

To further analyse the dynamic impact of fertility policy on the income gap between labour and capital, Eq (12) is constructed. $\sum_{i=1}^{n}\rho_{i}BirP_{ic(t-i)}$ in Eq (12) indicates that there may be a time lag effect on the impact of fertility policy on the income gap between labour and capital, and when *i* = *n*, it indicates that fertility policy still has a significant impact on the income gap between labour and capital with a lag of n periods. The empirical results are shown in Table 4 with *i* = *2*. According to the results in Table 4, although fertility policy with a lag of 2 periods has a significant effect on the income gap between labour and capital, the longer the lag, the weaker the effect.


$$LabS_{ict}=\beta_{0}+\beta_{1}BirP_{ict}+\sum_{i=1}^{n}\rho_{i}BirP_{ic(t-i)}+\beta_{2}Z_{ict}+\lambda_{i}+\lambda_{c}+\lambda_{t}+\varepsilon_{ict}$$ (12)

**Table 4:** Dynamic analysis results.

	(1)	(3)
*BirP*	-1.1322***	-0.7912***
	(0.0224)	(0.0241)
*BirP*_(t-1)_	-1.2361***	-0.0451**
	(0.0461)	(0.0221)
*BirP*_(t-2)_	-0.6822*	-0.0612**
	(0.0551)	(0.0321)
*TECH*		-0.0421***
		(0.0051)
*LTF*		0.0057**
		(0.0264)
*SF*		-0.0213***
		(0.0412)
*IR*		-0.3115***
		(0.0322)
*FEP*		0.0551**
		(0.0371)
*OE*		0.0612***
		(0.0372)
*C*	0.7731***	0.4691***
	(0.0422)	(0.0441)
observation number	23976	23976
*R* ^2^	0.6881	0.6481

Data source: calculated

Note: *, ** and *** denote 10%, 5% and 1% significance levels respectively; standard errors of coefficient estimates in parentheses.

### Validation of the assumption of closed economies

According to the theoretical mechanism of this paper, this study is in a relatively closed economy, which is to ensure that the majority of capital investment comes from savings in this economy. To highlight the correlation between household savings and social capital investment, an extreme case is assumed. If all capital investment by firms in the economy comes from outside the economy and internal savings are not used for investment, then savings that change as a result of a change in fertility policy will not be used for capital investment and thus will not affect the labour income share. Therefore, the less connected the economy is to the outside world, the stronger the ’one-to-one’ relationship between savings and capital investment, and the smaller the impact of external capital on the labour income share. Thus, the assumption that economies are closed enhances the extent to which fertility policy affects the labour-capital income gap.

To test the existence of this hypothesis, this paper compares the differences in the impact of fertility policies on the labour-capital income gap in closed versus open economies. The sample for this paper is 3015 domestic enterprises, including state-owned enterprises (including collective enterprises) and private enterprises, and 151 wholly foreign-owned enterprises, in 285 cities. The paper approximates those domestic enterprises represent closed economies, while foreign-owned enterprises represent open economies. The impact of fertility policies on labour income shares in the two economies is compared. According to the theory in this paper, the effect of fertility policy on the labour income share of wholly foreign-owned enterprises is small or even insignificant because most of the capital of such enterprises comes from abroad. In contrast, state-owned enterprises and private enterprises, where most of the capital is sourced domestically, should have a significant impact on the labour income share of these two types of enterprises. And, this effect is also large compared to that of wholly foreign-owned enterprises. The results of the test are shown in Table 3.

Columns (1) and (2) of Table 5 show that fertility policy has a negative impact on the labour income share of state-owned and private enterprises, consistent with the theoretical analysis; column (3) of Table 5, the impact of fertility policy on wholly foreign-owned enterprises is also consistent with the theoretical analysis. It should be noted that while the results in column (3) of Table 5 hold at the 10% level of significance, the coefficients have the smallest estimates and this effect is an order of magnitude smaller compared to state-owned and private enterprises. The results in Table 5 validate the closed economy requirement of the theoretical mechanism.

**Table 5:** Verification of closed economic hypothesis.

	state-owned enterprise	private enterprise	foreign-funded enterprise
	(1)	(2)	(3)
*BirP*	-0.0683***	-0.0664***	-0.0051***
	(0.0224)	(0.0113)	(0.0078)
*TECH*	-0.0225**	-0.0411***	-0.0352**
	(0.0032)	(0.0224)	(0.0381)
*LTF*	0.0061	0.0042**	0.0144***
	(0.0077)	(0.0115)	(0.0252)
*SF*	-0.0366	-0.0351**	-0.0026***
	(0.0557)	(0.0211)	(0.0044)
*IR*	-0.4088**	-0.3991***	-0.3758***
	(0.0503)	(0.0661)	(0.0255)
*FEP*	0.1152*	0.0548**	0.0885**
	(0.0483)	(0.0331)	(0.0562)
*OE*	0.1154***	0.2251***	0.3226***
	(0.0075)	(0.1172)	(0.0241)
*C*	0.5132***	0.3804**	0.2043***
	(0.0451)	(0.0032)	(0.0052)
observation number	8098	17932	1373
*R* ^2^	0.6681	0.6621	0.5622

Data source: calculated

Note: *, ** and *** denote 10%, 5% and 1% significance levels respectively; standard errors of coefficient estimates in parentheses.

### Test results of mediation mechanism

According to the theoretical mechanism of this paper, capital intensity is the mediator variable of fertility policy affecting labour income share. If an encouraging fertility policy is implemented, then households increase their household savings rate in order to raise offspring, bringing about an increase in social capital investment, which ultimately leads to a decrease in the labour income share. This mediating influence mechanism can be verified based on the Sobel-Goodman mediator variable test model, which is constructed according to Eqs (13)–(15). Where *CapI* is the capital intensity and the control variables are denoted by Ω、 Δ and Ψ in the three equations respectively. And, the sample was selected from 3015 domestic enterprises.


$$LabS_{ict}=\alpha_{0}+\alpha_{1}BirP_{ict}+\alpha_{2}\Omega_{ict}+\delta_{ict}$$ (13)

$$CapI_{ict}=\gamma_{0}+\gamma_{1}BirP_{ict}+\gamma_{2}\Delta_{ict}+\varsigma_{ict}$$ (14)

$$LabS_{ict}=\nu_{0}+\nu_{1}BirP_{ict}+\nu_{2}CapI_{ict}+\nu_{3}\Psi_{ict}+\omega_{ict}$$ (15)

The process of testing capital intensity as a mediator variable is as follows. First, Eq (13) tests the significance between fertility policy and labour income share. Secondly, if the test of the coefficient on fertility policy in Eq (13) is significantly non-zero, then Eq (14) is applied to test the significance of the relationship between fertility policy and capital intensity. Finally, if the coefficient on fertility policy in Eq (14) also turns out to be significantly non-zero, then Eq (15) is used to test the significance of the relationship between fertility policy, capital intensity and labour income share. If the coefficient on capital intensity is significantly non-zero, then the mediation mechanism holds. Since the results of Eq (13) are already given by column (3) of Table 2, which shows that fertility policy is significantly negative for labour income share, the test is not repeated. To highlight the scientific nature of the role of the mediator variable of capital intensity, Eq (16) is added, with Φ as the control variable, which is the same as Eq (10). Without the effect of fertility policy, if capital intensity has a significant effect on labour income share, then it verifies that the theoretical mechanism of capital intensity significantly affecting labour income share holds.


$$LabS_{ict}=\mu_{0}+\mu_{1}CapI_{ict}+\mu_{2}\Phi_{ict}+\eta_{ict}$$ (16)

Column (1) of Table 6 verifies Eq (16), the negative effect of capital intensity on the labour income share. In the model without the fertility policy variable, an increase in capital intensity by 1 decreases the labour income share by 0.0513. This implies that capital intensity has an impact on the labour income share. If fertility policy has an effect on capital intensity, then it further has an effect on labour income share. This suggests that capital intensity can be used as a mediator variable for fertility policy to affect labour income shares. Of course, Eq (16) holds only as a necessary condition, and the above mediation mechanism requires a rigorous verification process.

**Table 6:** Mediation mechanism test.

Dependent variable	Labour income share	*CapI*	Labour income share
Independent variable	(1)	(2)	(3)
*BirP*		0.3232***	-0.0612***
		(0.0043)	(0.0204)
*CapI*	-0.0497***		-0.1152***
	(0.0155)		(0.0065)
*TECH*	-0.0274***	-0.3551**	-0.0533**
	(0.0166)	(0.0047)	(0.0084)
*LTF*	0.0046***	0.0271***	0.0132**
	(0.0053)	(0.0122)	(0.0077)
*SF*	-0.0072**	0.3351***	-0.0142***
	(0.0031)	(0.0082)	(0.0057)
*IR*	-0.3772**	0.3481**	-0.3861**
	(0.0028)	(0.0043)	(0.0052)
*FEP*	0.0416***	-0.5302***	0.0644***
	(0.0221)	(0.0366)	(0.0048)
*OE*	0.0625***	-0.0561**	0.0503**
	(0.0781)	(0.0337)	(0.0074)
*C*	0.3604**	0.1132**	0.3711***
	(0.0024)	(0.0336)	(0.0067)
observation number	27403	27403	27403
*R* ^2^	0.6683	0.5504	0.6205

Data source: calculated

Note: *, **and ***denote 10%, 5% and 1% significance levels respectively; standard errors of coefficient estimates in parentheses.

The first step to verify that capital intensity is the mediating variable is in Eq (13), which has been verified by column (3) of Table 2, i.e. the effect of fertility policy on labour income share is significantly negative. The second three steps are shown in Eqs (14)–(15), which are verified in columns (2)-(3) of Table 6. Column (2) shows that fertility policy has a positive effect on capital intensity at the 5% level of significance. In column (3), the coefficient of the fertility policy variable is significantly zero, while the capital intensity variable is significantly non-zero at the 1% significance level. this indicates that capital intensity has a full mediating effect, i.e. fertility policy cannot affect the labour income share directly, but indirectly through capital intensity. The above analysis verifies that the mediating mechanism holds.

### Test results for group regression

China has had a strict ’one-child policy’ since 1980, which was gradually relaxed until 2014, when a family was initially allowed to have two children and then three children in 2021. According to the theoretical mechanism, the fertility policy variable changes significantly in 2014, with a significant increase in the number of children born to a family of two. significantly increased. If fertility policy has a significant effect on labour income shares, then the model before and after this policy change should be significantly different, i.e. it could be a group regression model. The regression results of substituting Eq (10) for the 2003–2013 sample and the 2014–2019 sample are shown in (1) and (2) of Table 7, respectively. The regression coefficient for the former is -0.0102 and for the latter is -0.1830 and both pass the 1% significance test.The Fisher’s test p-value results indicate that the regression result of -0.1830 for the period 2014–2019 is significantly different compared to the regression result of -0.0102 for the period 2003-2013.This suggests that, following the introduction of the two-child policy, households with a need to have children responded immediately, including by increasing their savings to cope with the birth and upbringing of a second child, so that the share of labour income decreased as the amount of capital investment increased.

**Table 7:** Test of threshold effect.

	2003–2013	2014–2019
	(1)	(2)
*BirP*	-0.0102***	-0.1830***
	(0.0226)	(0.0381)
*TECH*	-0.0414*	-0.0461**
	(0.0072)	(0.0251)
*LTF*	0.0513**	0.0336**
	(0.0052)	(0.0131)
*SF*	-0.0274***	-0.0381***
	(0.0036)	(0.0155)
*IR*	-0.3052**	-0.4105**
	(0.0063)	(0.0073)
*FEP*	0.0533**	0.0312***
	(0.0041)	(0.0152)
*OE*	0.0835***	0.1260***
	(0.0042)	(0.0204)
*C*	0.5204***	0.6034**
	(0.0103)	(0.0079)
observation number	17731	9672
*Fisher’s test p-value*	0.0080	
*R* ^2^	0.5302	0.6027

Data source: calculated

Note: *, **and *** denote 10%, 5% and 1% significance levels respectively; standard errors of coefficient estimates in parentheses.

This result not only validates the existence of a threshold effect mechanism, but also further validates the dampening effect of incentive-based fertility policies on the labour income share, which declined significantly after the easing of fertility policies in 2014. This suggests that, following the introduction of the two-child policy, households with a need to have children responded immediately, including by increasing their savings to cope with the birth and upbringing of a second child, so that the share of labour income decreased as the amount of capital investment increased.

## Conclusions

This paper examines the mechanisms by which changes in fertility policy affect the labour-capital income gap. In a closed economy, fertility policy has a dampening effect on labour income shares, with capital intensity as a mediating variable. And, the relaxation of the one-child fertility policy in 2013 creates a threshold. This paper tests the scientific validity of this theoretical mechanism by selecting a sample of 4795 firms in 285 prefectural-level cities across China, and the conclusions obtained are as follows.

First, the mechanism by which fertility policy affects the labour-capital income gap. Using the production function, it is shown that in relatively closed economies, incentivised fertility policies widen the labour-capital income gap. This is because incentive-based fertility policies increase households’ precautionary savings. The sum of the savings of all households in society constitutes total social capital, and its increase implies an increase in the amount of capital input in production. According to the principle of income distribution by factor contribution, the income of capital owners increases, while the income of labour decreases. Further, the robustness of the model is verified by excluding cities with a high concentration of ethnic minorities, excluding samples with large dispersion and replacing the dependent variable, respectively. And, the difference-in-difference model is also applied to prove that there is no endogeneity in Eq (10). Moreover, the analysis of the dynamics of the impact of fertility policies on the income gap between labour and capital shows that there is a time lag in this impact, which can be seen in Table 4, with a time lag of 2 periods.

Second, capital intensity is a mediator variable for fertility policy to affect the labour-capital income gap. As can be seen from Table 6, fertility policy affects the labour-capital income gap by using capital intensity as a mediator variable and with a full mediation effect. Fertility policy cannot directly affect the labour-capital income gap; it must do so through the mediator variable of capital intensity. The results of the three regressions in column (2) of Table 2 and column (2)(3) of Table 6 confirm the validity of this mediating mechanism. Moreover, column (3) of Table 6 further indicates that the mediation mechanism in this paper is a full mediating effect.

Third, the impact of fertility policies on the labour-capital income gap is analysed in a closed economy. The study in this paper focuses on a relatively closed economy, excluding the effect of foreign investment. This is because most foreign-owned enterprises are financed from outside the country and are not associated with domestic savings, and are therefore less affected by domestic fertility policies. Table 5 validates this mechanism by comparing the regression results for domestic firms with those for wholly foreign-owned firms. While the effect of fertility policy on the labour-capital income gap among wholly foreign-owned firms is also significant, the significance level requires 10%, which is higher than the 1% for domestic firms. This suggests that the statistical rejection of the probability of a correlation between the two is 10 times higher for wholly foreign-owned firms than for domestic firms, and the regression coefficient for the former is also much lower than for the latter. Thus, the effect of fertility policy on the labour-capital income gap is more pronounced in closed economies.

Fourthly, the threshold effect of fertility policies affecting the gap between labour and capital income. China implemented a strict one-child family planning policy from 1980 until 2013, when the policy was relaxed. Therefore, there is a threshold effect of the impact of fertility policy on the labour-capital income gap around 2013. Table 7 shows that there is a threshold at which fertility policy affects the labour-capital income gap, and that the threshold corresponds to 2013. Moreover, after the threshold, the negative impact of fertility policy on the labour-capital income gap more than doubles. Therefore, the threshold effect holds.

## Implications

The key to narrowing the labour income gap widened by fertility policies is to reduce the share of precautionary savings in total income, especially for low-income households. Therefore, on the one hand, it is important to enhance the human capital of low-income households to create human capital bonus and raise total household income. China’s fertility policy has been gradually adjusted from the strict "one-child" policy to the more lenient " universal two-child" policy, and the "three-child" policy has been implemented in 2021 to better adapt to the new changes in the demographic situation and the new challenges of the population. This series of changes in fertility policy will undoubtedly help to achieve an appropriate level of fertility, optimise the population structure, and ease the pace of population ageing and the burden on social security; however, it should also be noted that the "three-child" policy is likely to affect mainly low-human-capital families, which may have a negative impact on the increase in average human capital and the fairness of income distribution in society, and there is an urgent need to improve relevant measures. Therefore, the implementation of the "three-child" policy should be accompanied by a series of support measures aimed at improving the quality of the population and reducing the costs of childbearing. The relevant economic and social support policies should be further optimised and improved, so as to stabilise the reasonable fertility level and at the same time comprehensively improve the quality of the population and create a good "human capital dividend". In terms of China’s long-term demographic and economic and social development trends, the abolition of the birth control policy may be inevitable in the future, which highlights the importance of optimising and improving the relevant supporting policies in order to create a "human capital dividend" and increase the total income of families.

On the other hand, the total amount of household precautionary savings should be reduced. Chinese families’ precautionary savings are mainly used for education, serious illnesses and property purchases. Among them, education has become one of the most important burdens for Chinese families because of its certainty and durability. Therefore, the reduction of preventive savings of Chinese families should start from the burden of family education. While strictly implementing the "double reduction" policy (i.e., reducing the burden of homework and out-of-school training for students in the compulsory education stage), China should continue to increase public investment in education, especially in rural areas and underdeveloped regions, and make efforts to realise equity in education, so as to provide a good guarantee for the accumulation of human capital for human-capital-poor families, and prevent them from falling into the human-capital accumulation trap. from falling into the human capital accumulation trap. At the same time, the Chinese government should actively take strong measures to enhance social mobility, create a fairer competitive environment, effectively stimulate the endogenous impetus and initiative of human capital accumulation in the whole society, and actively create human capital dividends. While promoting economic growth, fair income distribution and easing the pressure of the social burden of old-age pensions, the Chinese Government should achieve high-quality, sustainable and coordinated development of the population and the economy and society. The main ways to reduce the burden of major diseases are healthcare reform and the purchase of commercial medical insurance, so it is particularly important for China to reform its healthcare system and design effective commercial insurance for healthcare. Chinese society should promote good marriage customs, stop the phenomenon of "marriage tournaments", reduce the cost of marriage, and reduce precautionary family savings.

## Policy suggestion

While increasing the level of labour income and reducing precautionary savings are two paths to narrowing the labour-income gap, the latter path is more feasible. In fact, China has introduced a number of policies to reduce precautionary savings in order to narrow the labour income gap. For example, the personal income tax credit policy has helped low- and middle-income families bear some of the costs of education, health care, and housing. Also, the duration of parental leave has been extended. And, for each additional child, the leave is extended by 90 days. And, there are also maternity leave paternity measures of varying lengths in various regions. However, these policies are not strong enough. This paper recommends the introduction of policies to design effective commercial insurance, the formation of policy financial institutions and a moderate increase in the number of wholly foreign-owned enterprises to reduce precautionary savings.

Formation of policy financial institutions that provide financial support for micro and small enterprise loans, especially those that provide financial support for entrepreneurship. Although entrepreneurship is risky, it is also an effective way to mitigate the high cost of feeding families with many children. Although the Chinese government encourages financial institutions to provide loans to micro and small enterprises, the amount is limited, so entrepreneurial families need to save a lot of money. Even if they get a loan to start a business, they will face high financing costs because of the high risk of starting a business, so household savings are the best way to cope with these. Because of the high risk of entrepreneurship, the wealth accumulated from entrepreneurship is used more for savings to increase risk resilience. If external financing is needed, then the financing is also conditional on household assets or savings, so loans for entrepreneurship are also contingent on high household savings. Moreover, innovative households tend to be adept at financial management and have a stronger willingness to save. It follows that the high savings of entrepreneurial households are a result of the difficulty of obtaining credit support when funds may be scarce at any time. Therefore, we should set up a policy-oriented financial institution specialised in serving " public entrepreneurship". This will not only increase the success rate of entrepreneurial households and help to create highly innovative enterprises, but will also help to reduce the total amount of social savings and thus reduce labour income inequality.

In addition, based on the assumption of a closed economy, the introduction of offshore capital investment is also an effective way to reduce the inequality between labour and capital incomes. Since foreign capital investment is not related to domestic savings, the more foreign investment there is, the more employment there is for domestic labour, which naturally raises workers’ incomes. Although foreign capital also receives a corresponding share of the remuneration, since the capital gains do not belong to China, it does not change China’s share of capital income, but at the same time raises the share of labour income, thus reducing the income inequality between labour and capital. It should be noted, however, that while the introduction of foreign capital into China has helped to reduce the labour-capital income gap, some literature suggests that it has raised skill and gender wages, and also widened the urban-rural income gap. In view of this, the use of foreign capital to reduce the income gap between labour and capital should also take into account changes in other income gaps and determine a reasonable amount of foreign capital to be introduced.

## Future research

Income equity helps to maintain social stability, stimulate economic vitality, and enhance scientific and technological innovation, so the study of factors affecting income disparity has certain academic value. China’s fertility policy has changed many times in the past 10 years, which has had a significant impact on the income gap. In view of this, the impact of changes in fertility policy on income gap is worth studying. It should be noted that according to the classification of the sources of income gap, the income gap includes the urban-rural income gap, the income gap between regions, the income gap between labour and capital, the income gap between industries, the income gap between different skills and so on. Since the mediating variable in this paper is capital accumulation, this study focuses on the income gap between labour and capital and does not address other income gaps. Therefore, the subsequent research focuses on the impact of fertility policies on other types of income gaps and forms a complete research system on this topic.

## Supporting information

S1 Dataset Raw data used in this paper. (XLSX)
